# *Glechoma hederacea* (L.): Phytochemical Profile, Biological Activities and Emerging Perspectives in Oral Health—A Review

**DOI:** 10.3390/plants15152396

**Published:** 2026-08-05

**Authors:** Alicia-Denisa Bereteu-Costa, Annamaria Pallag, Georgiana Ioana Potra Cicalău, Mariana Ganea, Manuela Bianca Pașca, Monica Tabita Morar, Gabriela Ciavoi

**Affiliations:** 1 Doctoral School of Biological and Biomedical Sciences, University of Oradea, Universitatii Str. No 1, 410087 Oradea, Romania; costa.aliciadenisa@student.uoradea.ro (A.-D.B.-C.); morar.monicatabita@student.uoradea.ro (M.T.M.); 2 Department of Pharmacy, Faculty of Medicine and Pharmacy, University of Oradea, P-ta 1 Decembrie No 10, 410073 Oradea, Romania; mganea@uoradea.ro (M.G.); bpasca@uoradea.ro (M.B.P.); 3 Department of Dental Medicine, Faculty of Medicine and Pharmacy, University of Oradea, 10 1st Decembrie Street, 410073 Oradea, Romania; gciavoi@uoradea.ro

**Keywords:** *Glechoma hederacea*, medicinal plants, phytochemistry, antioxidants, antimicrobial activity, oral health, dentistry, flavonoids

## Abstract

Current interest in the use of medicinal plants for the prevention and management of various diseases has increased significantly due to the need to identify effective, safe, and sustainable natural agents. *Glechoma hederacea* L., a perennial species belonging to the *Lamiaceae* family, has traditionally been used in European folk medicine for the treatment of respiratory, inflammatory, and digestive disorders. Modern research has highlighted a complex phytochemical profile dominated by phenolic acids, flavonoids, and volatile compounds that have been associated with a variety of biological activities. Experimental studies have reported antioxidant, antimicrobial, anti-inflammatory, and cytoprotective effects. The present review summarizes current data regarding the phytochemical composition and biological properties of *Glechoma hederacea*, with particular emphasis on its potential applications in oral hygiene products and complementary dental therapy.

## 1. Introduction

Nature has always been an essential source of compounds with therapeutic potential, providing numerous medicinal plants rich in phytochemicals with significant biological value [1].

Phytotherapy, defined as the therapeutic use of medicinal plants, plays an important role in the prevention, management, and treatment of numerous diseases worldwide [2,3,4].

The *Lamiaceae* family represents one of the largest botanical families, encompassing numerous plant species with diverse biological activities, which is why they are frequently used in medicinal practice [5]. This family is particularly recognized for its aromatic and culinary plants, such as oregano, thyme, sage, wild thyme, mint, basil, rosemary, and lemon balm. In addition, the *Lamiaceae* family includes species with underexplored therapeutic potential that have long been used in traditional medicine but remain insufficiently investigated in terms of their phytochemical composition and bioactive properties [6,7,8,9,10,11].

*Glechoma hederacea* L., a plant belonging to the *Lamiaceae* family, is commonly known as “ground ivy” or “creeping charlie”. Available experimental studies have reported that *Glechoma hederacea* extracts and several of their phytochemical constituents exhibit a wide range of biological activities [12]. In traditional Chinese medicine, it is used for the treatment of diabetes, gallstones, inflammation, water retention, and abscesses. In addition, it exerts beneficial effects on the immune, respiratory, and urinary systems. Traditional use and experimental studies have suggested beneficial effects on skin and hair health, as well as antibacterial and antifungal activities [13]. Plants belonging to the genus *Glechoma*, within the *Lamiaceae* family, have long been used in both Asian medicine and Western healthcare systems, being recognized for their therapeutic properties [13,14,15,16,17,18]. Although *Glechoma hederacea* is not widely recognized as a medicinal plant in the scientific literature and its therapeutic effects have been insufficiently investigated, it has been used in traditional medicine in northwestern Romania since the time of the Dacians [19,20]. The dried herb of *Glechoma hederacea* is valued both for its therapeutic effects and as a food product available on the market, the best-known example being gill tea. The reported pharmacological activities of *Glechoma hederacea* extracts are believed to be largely associated with their high content of phenolic compounds, particularly phenolic acids and flavonoids, which are present in significant amounts in this plant species [13,21,22]. Among the most important phenolic compounds identified in *Glechoma hederacea* are phenolic acids such as rosmarinic, protocatechuic, caffeic, chlorogenic, cryptochlorogenic, and ferulic acids [20,23]. In addition, flavonoids such as rutin, isoquercetin, genistin, genistein, and daidzin have also been identified in *Glechoma hederacea*. The available evidence suggests that this plant species possesses promising pharmacological properties [6]. In recent years, natural compounds with anti-inflammatory and bone-protective effects have become an important research topic in periodontal therapy. Experimental studies have reported that *Glechoma hederacea* may inhibit osteoclast differentiation and bone resorption by interfering with molecular mechanisms involved in NFATc1 activation, suggesting a potential role in the management of periodontitis [24]. Periodontitis is a chronic non-communicable inflammatory disease that affects the structures of the periodontium and leads to their irreversible destruction [25]. The progression of the disease is generally slow and accompanied by mild or nonspecific symptoms, which often go unnoticed by patients or are not associated with periodontal disease. As a result, many individuals seek dental care only in advanced stages of the disease, when complex therapeutic interventions are required [26]. Periodontitis causes the progressive destruction of the tooth-supporting structures and, if left untreated, may ultimately lead to tooth loss [27]. The pathogenesis of this condition is influenced by multiple factors, including the host immune response, genetic predisposition, and lifestyle-related factors such as smoking, stress, and diabetes, all of which may contribute to increased disease severity [28]. Chronic inflammation can disrupt the balance of bone remodeling processes by excessively stimulating osteoclast formation and activity, thereby promoting conditions associated with bone tissue destruction. In periodontitis, the persistent immune response triggered by the excessive accumulation of microorganisms within the periodontium leads to inflammation of the gingiva and periodontal ligament [29]. As the inflammatory process progresses, numerous pro-inflammatory cytokines are released, stimulating osteoclast activity and contributing to alveolar bone resorption [13,21,24,30]. The aim of this review is to summarize the current evidence regarding the phytochemical profile and biological properties of *Glechoma hederacea*, with particular emphasis on its applications and emerging relevance in oral health. Although previous reviews have comprehensively addressed the botanical characteristics, phytochemistry, and general pharmacological properties of *Glechoma hederacea*, no review has specifically focused on its relevance to oral health. Therefore, the present review evaluates the available evidence from a dental perspective, distinguishes between direct oral evidence and indirect experimental findings, and identifies current knowledge gaps and future research directions for future applications of *Glechoma hederacea* in oral healthcare.

## 2. Methodology

This study was conducted as a narrative review based on a structured search of the available scientific literature concerning *Glechoma hederacea* L. To this end, an extensive literature search was conducted, from which studies considered relevant to the topic were selected. No restrictions regarding the year of publication were applied; therefore, both recent articles and older studies were included to ensure the most comprehensive coverage of the information available in the literature. The primary sources consulted were the Web of Science Core Collection and Scopus databases, selected for their access to high-quality studies addressing the biological activity and therapeutic potential of *Glechoma hederacea* L.

To identify scientific articles available up to the date of the literature search, a bibliographic search was performed using relevant keywords to retrieve primary data, experimental findings, and existing literature in the field. The search strategy included terms such as “*Glechoma hederacea*”, “phytochemical composition”, “anti-inflammatory activity”, “antimicrobial activity”, “oxidative stress”, “medicinal plants”, “periodontitis” and “oral inflammation” in order to identify studies relevant to the subject under investigation. Original articles, in vitro and in vivo experimental studies, and review papers published in peer-reviewed scientific journals were included in this analysis. Sources lacking scientific validation, articles without full-text availability, duplicate publications, and studies not directly related to the biological or pharmacological activity of the plant were excluded.

**Figure 1:**
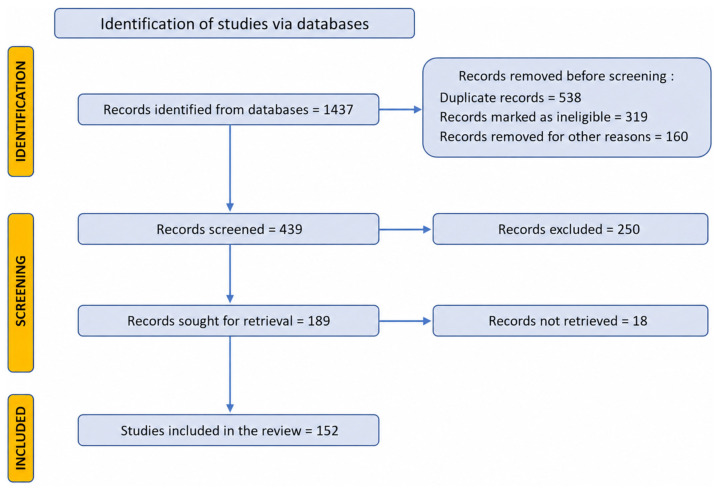
Flow diagram illustrating the literature search and study selection process for the present narrative review.

The data extracted from the selected literature were organized and qualitatively analyzed according to several categories, including the phytochemical composition of the plant, antioxidant effects, anti-inflammatory activity, antimicrobial activity, and their relevance to oral health. The interpretation of the findings also considered the study design and the type of experimental evidence reported in the selected publications. Due to the methodological heterogeneity of the included studies, a narrative synthesis of the findings was conducted. The literature identification and study selection process is summarized in Figure 1 to improve the transparency and reproducibility of the structured literature search conducted for this narrative review. The electronic literature search was performed between 15 February and 31 July 2026. The structured search identified 1437 records, of which 152 studies fulfilled the predefined eligibility criteria and were included in the qualitative synthesis presented in this review.

## 3. Taxonomy and Botanical Description of *Glechoma hederacea* L.

### 3.1. Taxonomy

*Glechoma hederacea* belongs to the *Lamiaceae* family, one of the most important families of medicinal plants, characterized by a high content of volatile oils and bioactive phenolic compounds.

The taxonomic classification is as follows:

- Kingdom: *Plantae*;
- Phylum: *Tracheophyta*;
- Class: *Magnoliopsida*;
- Order: *Lamiales*;
- Family: *Lamiaceae*;
- Genus: *Glechoma*;
- Species: *Glechoma hederacea* L. [31,32].

### 3.2. Botanical Description

*Glechoma hederacea* is a perennial species with a stoloniferous growth habit, exhibiting a high capacity for vegetative propagation. The stems are quadrangular, a characteristic feature of the *Lamiaceae* family, and may be either creeping or ascending, reaching lengths of approximately 10–50 cm.

The leaves are arranged oppositely and range from reniform to cordiform in shape, with crenate margins, generally reaching a diameter of 2–4 cm. When crushed, they release a characteristic aromatic odor due to their volatile oil content.

The flowers are small and bilabiate, typical of the *Lamiaceae* family, with coloration varying from blue-violet to lilac. They are arranged in groups of 2–3 within the leaf axils, and the flowering period generally extends from spring to early summer (April–June).

**Figure 2:**
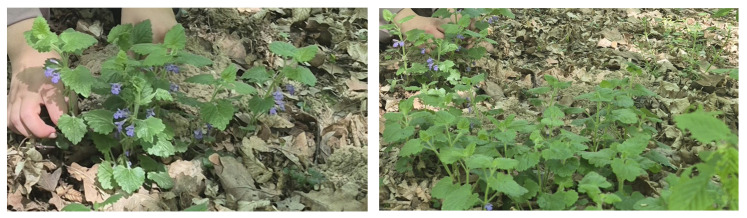
*Glechoma hederacea* L. in its natural habitat. Representative photographs of *Glechoma hederacea* showing the characteristic morphology of the species, including the creeping growth habit, reniform leaves, and blue-violet flowers. Photographs were taken in Băile Felix, Bihor County, Romania, on 16 April 2026. Images are the property of the authors. Botanical identification was performed using standard taxonomic keys and verified according to Plants of the World Online. No voucher specimen was prepared for the photographed material.

The fruit is a nutlet composed of four small brown units. Seed dispersal occurs through mixed mechanisms, predominantly by gravity and through insect-mediated dissemination [33]. The characteristic morphology of *Glechoma hederacea* is illustrated in Figure 2.

## 4. Active Compounds

Phenolic compounds represent the main bioactive constituents of *Glechoma hederacea* and are presented in Table 1. In addition, the ground plant material contains significant amounts of essential oils, vitamin C, provitamin A, as well as minerals such as zinc, iron, silicon, molybdenum, and calcium [34,35,36,37].

It should be noted that many biological activities described in the following sections originate from studies investigating isolated phytochemicals rather than whole *Glechoma hederacea* extracts. Consequently, the biological effects of individual compounds should not be interpreted as being directly equivalent to those of the complete plant extract, whose activity may be influenced by complex phytochemical interactions. Furthermore, findings reported for related *Glechoma* species are discussed only to provide supportive context and should not be interpreted as direct evidence for *Glechoma hederacea*.

Polyphenols constitute a major class of natural compounds that have been extensively investigated because of their diverse biological activities and potential health benefits [38,39]. These compounds are found in numerous plant-derived foods, including vegetables, cereals, and fruits, as well as in various beverages and food products such as teas and spices [40]. Based on their chemical structure, phenolic acids can be classified into several distinct categories. Hydroxycinnamic acids, characterized by a nine-carbon skeleton, include a series of cinnamic acid derivatives such as *p*-coumaric acid, caffeic acid, ferulic acid, chlorogenic acid, and sinapic acid. In contrast, hydroxybenzoic acids, which possess a seven-carbon structure, are derived from benzoic acid and include compounds such as gallic acid, salicylic acid, protocatechuic acid, ellagic acid, gentisic acid, and syringic acid. Furthermore, phenolic acids may occur either in free form or bound to various molecular structures, including simple sugars, organic acids, or plant-derived polymers [41,42].

Several studies have investigated the phytochemical composition of *Glechoma hederacea* and consistently identified phenolic acids as the predominant bioactive constituents, although differences in extraction methods and analytical approaches have resulted in some variability in the reported phytochemical profiles. Representative studies are summarized below.

**Table 1:** Main phenolic acids identified in the phytochemical composition of *Glechoma hederacea* [43,44,45].

Phenolic Acids Identified in *Glechoma hederacea*		
Bioactive Compound/Chemical Structure (Schematic)		
Rosmarinic acid	Caffeic acid	Chlorogenic acid
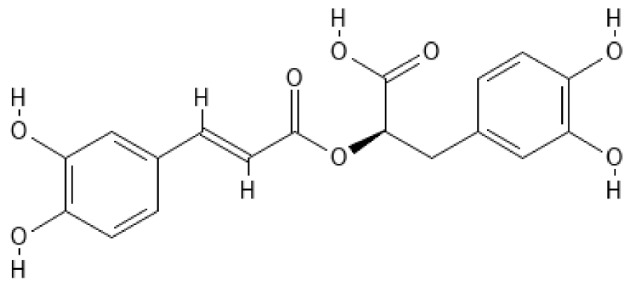	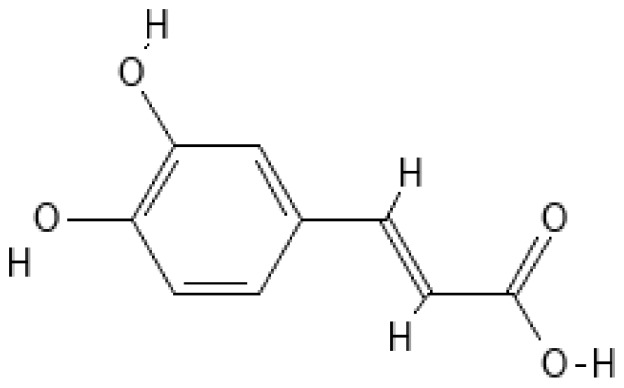	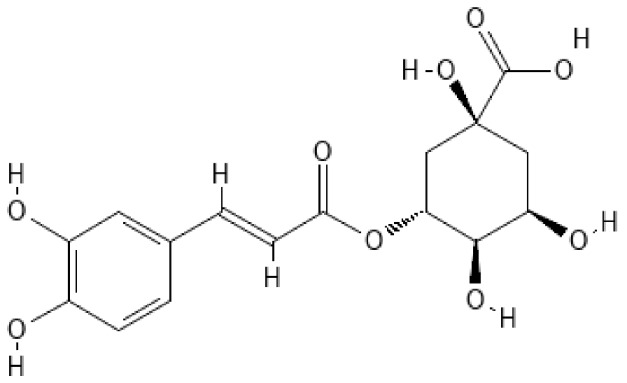
[46]	[47]	[48]
Ferulic acid	*p*-Coumaric acid	
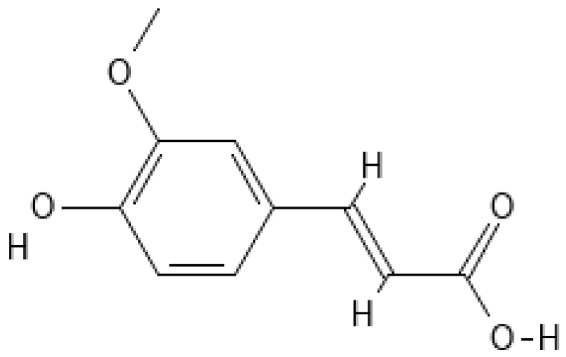	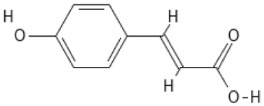	
[49]	[50]	

The phenolic profile and antioxidant activity of *Glechoma hederacea* extracts were investigated in a study conducted by Weronika Walendziak in 2025, which investigated phenolic acids such as chlorogenic, rosmarinic, caffeic, and ferulic acids using aqueous and ethanol-based extracts that demonstrated significant antioxidant activity [51,52].

A cell-based study conducted by Su-Tze Chou reported that rosmarinic acid, chlorogenic acid, caffeic acid, and ferulic acid exhibit significant antioxidant and anti-inflammatory activities, with rosmarinic acid being regarded as one of the most important polyphenols. Furthermore, the study revealed the antimicrobial and anti-inflammatory effects of rosmarinic acid identified in *Glechoma hederacea* extracts [9,53].

Rosmarinic acid is a bioactive phenolic compound commonly found in plants belonging to the *Lamiaceae* and *Boraginaceae* families [54]. From a biosynthetic perspective, it is formed from the amino acids tyrosine and phenylalanine through a series of enzymatic reactions. Alternatively, in chemical synthesis, RA is produced through the esterification of caffeic acid with 3,4-dihydroxyphenyllactic acid, a structure that contributes to the presence of the two phenolic rings characteristic of the molecule. Numerous experimental studies have reported that rosmarinic acid exhibits a broad spectrum of biological activities and has been investigated for its potential role in the management of several conditions, including cancer, diabetes, inflammatory diseases, neurodegenerative disorders, and liver diseases. Previous studies have also suggested its potential as an antiplasmodial, antiviral, and antibacterial agent [55,56,57]. Due to its strong antioxidant activity, this natural polyphenol has attracted increasing interest in recent years and is considered a potential nutraceutical compound with applications in the food industry. Overall, available evidence suggests that rosmarinic acid possesses a complex biological profile and multiple biomedical implications, with considerable potential for maintaining human health and for various therapeutic applications [13,55]. Rosmarinic acid has also been reported to exert protective effects in other inflammatory conditions, including periodontal disease, pancreatitis, neuropathy, ischemia, and allergic rhinitis. In gingival fibroblasts, RA-based treatments reduced the expression of the pro-inflammatory cytokines IL-1β, IL-6, and TNF-α, while also inhibiting inducible nitric oxide synthase. These findings suggest that modulation of the inflammatory response and improvement of antioxidant status represent proposed mechanisms that may contribute to slowing the progression of periodontitis [13,55,58,59].

Caffeic acid is naturally present in various dietary sources, including coffee beans, tea leaves, oats, rice, fruits, as well as argan oil and olive oil. Numerous studies, both in vitro and in vivo, have reported a wide range of important biological effects of this compound, including antioxidant, anti-inflammatory, antibacterial, antiviral, anti-atherosclerotic, anticancer, immunomodulatory, antidiabetic, cardioprotective, and hepatoprotective activities [60]. These effects are closely related to the ability of caffeic acid to influence inflammatory processes and oxidative stress within the body. Its antioxidant activity is mainly attributed to its capacity to neutralize reactive oxygen species. Given that oxidative stress is involved in the onset and progression of numerous diseases, the neutralization of free radicals may significantly contribute to their prevention and amelioration [13,61,62].

Chlorogenic acid is a natural polyphenol present in significant amounts in numerous plants, particularly in green coffee beans. Due to its bioactive nature, this compound has been reported to exhibit a variety of biological activities in different pathological contexts, addressing multiple forms of biological imbalance [63,64]. The chlorogenic acid family comprises a group of phenolic compounds abundantly present in plants, derived from the conjugation of the hydroxyl group of quinic acid with the carboxyl group of caffeic acid. This class includes 1L-(−)-quinic acid, caffeic acid, ferulic acid, as well as *p*-coumaric acid derivatives, including caffeoylquinic and feruloylquinic acids. These compounds have been associated with numerous beneficial effects and are involved in protecting the body against chronic inflammatory and age-related disorders, primarily through their anti-inflammatory and antioxidant activities [65,66,67,68,69,70,71]. Chlorogenic acid also exhibits a favorable safety profile, as it has not been associated with significant adverse effects or toxicity toward normal cells and tissues. Moreover, it is considered well tolerated in humans [72].

Ferulic acid is a phenolic compound widely distributed throughout the plant kingdom and represents an important active constituent of numerous traditional Chinese medicine preparations. To date, studies have shown that it exhibits multiple biological activities, being involved in the modulation of oxidative stress, inflammatory processes, vascular endothelial injury, fibrosis, apoptosis, and platelet aggregation. Furthermore, research indicates that ferulic acid may exert its effects through inhibition of the PI3K/AKT signaling pathway, reduction in reactive oxygen species production, and decreased aldose reductase activity [73,74,75,76]. Ferulic acid, a phenolic compound commonly found in plant cell walls, has also been shown to disrupt quorum sensing systems in several bacterial species [77].

*p*-Coumaric acid occurs in both free and esterified forms and is present in numerous fruits, vegetables, and grasses. This compound has attracted considerable scientific interest due to its antioxidant and chemoprotective properties. Over the years, numerous studies have focused on the extraction and transformation processes of *p*-coumaric acid. However, the exact mechanism underlying its antibacterial activity has not yet been fully elucidated [78]. In a study investigating the antibacterial activity of *p*-coumaric acid, the compound was tested against both Gram-positive and Gram-negative pathogenic bacteria. The study reported the strongest antimicrobial activity against *S. dysenteriae,* for which the minimum inhibitory concentration was 10 μg/mL. The mechanism of action was investigated through membrane permeabilization assays and bacterial DNA interaction studies [56,79,80]. Based on the obtained results, the authors concluded that *p*-coumaric acid causes the destruction of the pathogenic *S. dysenteriae* strain by inducing irreversible permeability of the bacterial cell membrane [81,82]. Research has reported that *p*-coumaric acid and its derivatives exhibit numerous bioactive properties, including antioxidant, antimicrobial, anticancer, anti-inflammatory, antiarthritic, antidiabetic, and antiangiogenic effects. In addition, this compound has been associated with gastroprotective, hepatoprotective, cardioprotective, and renoprotective activities, as well as with the stimulation of skin regeneration and bone formation [83]. Due to this broad bioactive potential, *p*-coumaric acid could be utilized in the development of food and pharmaceutical products [84]. Flavonoids represent a widely distributed class of natural polyphenolic compounds found in plants, generally in glycosylated forms. These compounds have been associated with a variety of biological activities, including antioxidant, anti-inflammatory, antibacterial, antiviral, and anticancer effects. The main flavonoids identified in *Glechoma hederacea* are presented in Table 2.

From a structural perspective, flavonoids are functional aromatic compounds characterized by a C6–C3–C6 carbon skeleton. Their biosynthesis begins with the amino acid L-phenylalanine, which is converted into phenylpropenoyl-CoA through a process catalyzed by the enzyme phenylalanine ammonia-lyase [85,86]. Due to their broad spectrum of biological activities, natural flavonoids have attracted increasing interest in the medical field. Consequently, numerous synthetic analogues are currently regarded as promising candidates for the treatment of conditions such as cancer, bacterial, fungal, and viral infections, as well as inflammatory diseases [87].

**Table 2:** Main flavonoids identified in *Glechoma hederacea* [38,43,88,89,90,91,92].

Flavonoids and Acids Identified in *Glechoma hederacea*		
Bioactive Compound/Chemical Structure (Schematic)		
Rutin	Quercetin	Apigenin
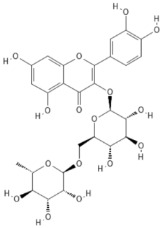	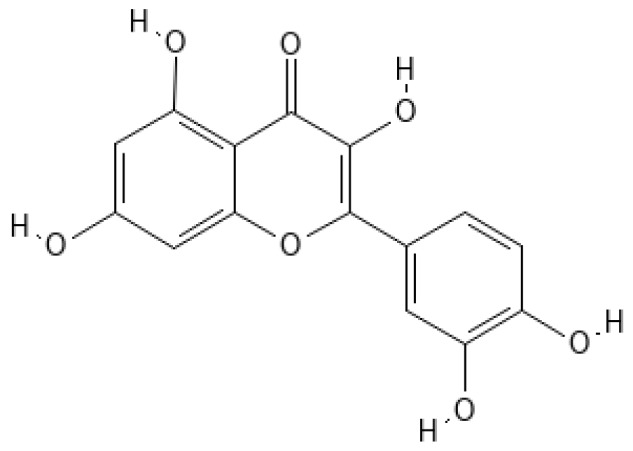	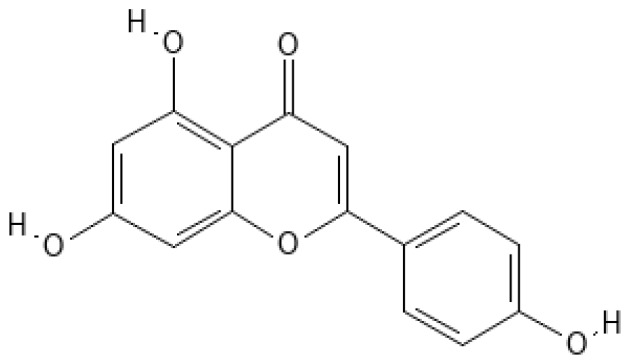
[93]	[94]	[95]
Luteolin derivatives	Kaempferol	
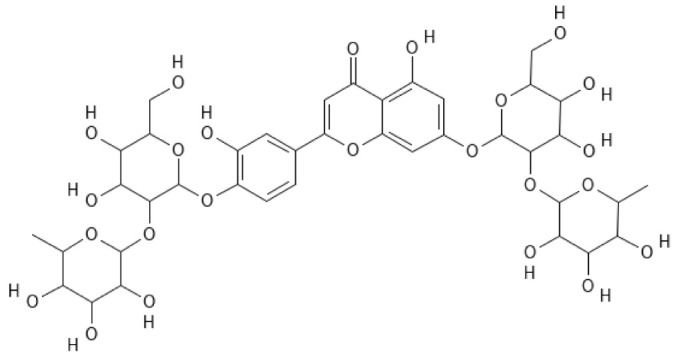	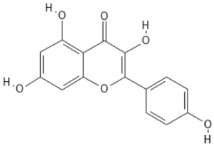	
[96]	[97]	

Among flavonoid compounds, flavones and flavonols are closely related, sharing the same fundamental structural framework, namely the 2-phenylchromen-4-one system. Flavones represent one of the most extensively studied subclasses of flavonoids due to their widespread distribution throughout the plant kingdom and their considerable structural diversity [12,98,99,100,101]. The principal phytochemical constituents identified in *Glechoma hederacea* and their reported biological activities are summarized in Table 3.

Among the principal compounds identified in *Glechoma hederacea* are rosmarinic acid, rutin, and derivatives of quercetin, luteolin, and apigenin, which have been reported to contribute to the modulation of oxidative stress and the modulation of inflammatory processes. In addition, triterpenoids such as ursolic acid and oleanolic acid are thought to contribute to the biological activity of the species through their anti-inflammatory, antimicrobial, and cytoprotective effects [12,13,36,100,110,111,112,113].

Overall, the phytochemical profile of *Glechoma hederacea* is characterized by a predominance of phenolic acids and flavonoids, with rosmarinic acid consistently reported as one of the major bioactive constituents. Although the available studies consistently identify phenolic compounds as the principal contributors to the biological activities of the species, differences in extraction solvents (e.g., aqueous, ethanolic, hydroethanolic, or hot-water extracts), the plant material investigated (whole extracts or aerial parts), and the analytical methods used for phytochemical characterization contribute to variability in the reported phytochemical profiles and biological activities. However, this methodological heterogeneity limits direct comparison among studies and highlights the need for standardized extraction and phytochemical characterization protocols. Most of the available studies investigated crude aqueous, ethanolic, hydroethanolic, or hot-water extracts, whereas standardized extracts with a defined phytochemical composition have rarely been evaluated. Consequently, variations in extract composition should be considered when interpreting and comparing the reported biological activities.

## 5. Medicinal Properties of *Glechoma hederacea*

The available evidence indicates that the pharmacological activities of *Glechoma hederacea* result from the combined action of multiple phytochemical classes rather than from a single bioactive constituent. The following studies collectively illustrate the diverse biological effects reported for the species, although differences in experimental design should be considered when interpreting the results.

A review study dedicated to *Glechoma hederacea* examined the botanical characteristics, phytochemical composition, ethnobotanical uses, and pharmacological activities of this plant. The authors highlighted that most of the studies conducted to date have focused on the therapeutic potential of the plant in inflammatory and urolithic disorders. Although the mechanisms underlying its biological effects have not yet been fully elucidated, they appear to be mainly associated with the presence of terpenoids, flavonoids, and polyphenolic compounds. In addition, other important pharmacological activities have been reported, including antimicrobial, antioxidant, and depigmenting effects [20,113]. Grabowska and colleagues investigated in vitro the cytotoxic properties of aqueous and ethanolic extracts obtained from the dried aerial parts of *Glechoma hederacea* at concentrations ranging from 10 to 100 μg/mL. Cytotoxicity was evaluated using two colorectal cancer cell lines (Caco2 and HT29) and two melanoma cell lines (HTB140 and A375). HepG2 hepatoma cells, considered a model with a phenotype similar to that of normal hepatocytes, as well as normal human HaCaT keratinocytes, were also included. The MTT assay indicated that the extracts did not exhibit toxic effects on either normal human cells or the investigated tumor cell lines. Furthermore, the extracts showed significant antioxidant activity, which was correlated with their chemical composition. In the study by Grabowska et al., the aqueous extract exhibited significantly higher antioxidant activity than the ethanolic extract under the extraction conditions investigated [17,36,114]. These findings illustrate how extraction solvent may substantially influence both the phytochemical profile and the biological activity of *Glechoma hederacea* extracts. Milovanovic and co-workers investigated the antioxidant potential of *Glechoma hederacea* with the aim of evaluating its suitability as a food additive. The authors reported that ethanol–water extracts (8:2, *v*/*v*) and ethyl acetate-purified extracts exhibited significantly stronger antioxidant activity compared with other types of extracts tested, as well as with commonly used commercial antioxidants such as tocopherol mixtures and butylated hydroxyanisole [115,116]. These observations further emphasize that extraction methodology influences the recovery of bioactive constituents and, consequently, the antioxidant activity reported across studies. Recently, Kim and colleagues isolated several terpenoids from *Glechoma hederacea* and reported a wide range of biological activities. These isolated terpenoids exhibited cytotoxic effects against human tumor cell lines, including ascitic ovarian carcinoma (SK-OV-3) and cutaneous melanoma (SK-MEL-2). Moreover, some of the terpenoids showed the ability to inhibit nitric oxide production, significantly stimulate nerve growth factor secretion in C6 glioma cells, and exert neurotrophic effects [107]. Another study reported that the extract, as well as three fractions isolated from the aerial parts of GH, was capable of reducing hydrogen peroxide-induced oxidative DNA damage in peripheral blood mononuclear cells [104]. Flavonoids are widely recognized for their antioxidant properties and have been reported to modulate multiple cellular processes through the neutralization of reactive oxygen species [105].

Gwiazdowska and collaborators evaluated the antimicrobial activity of a ground *Glechoma hederacea* extract obtained through supercritical CO_2_ extraction. The study reported the strongest antibacterial activity against *Staphylococcus aureus*, with a minimum inhibitory concentration of 0.3 mg/mL. Furthermore, the study showed that Gram-positive bacteria were more susceptible than Gram-negative bacteria [117]. Based on available in vitro studies, bioactive compounds identified in *Glechoma hederacea* extracts have been reported to have antimicrobial activity against pathogenic bacteria such as *Escherichia coli* and *Staphylococcus aureus*. These results support the potential use of the extract as a natural preservative agent in the food industry [102]. In another study, *Glechoma hederacea* was investigated with respect to its chemical composition and antioxidant and anti-inflammatory potential. The aim of the research was to evaluate the aqueous extract obtained by hot-water infusion (HWG), both in terms of its phytochemical profile and biological activities.

The results revealed that the extract predominantly contained compounds such as rosmarinic acid, chlorogenic acid, caffeic acid, rutin, genistin, and ferulic acid. Antioxidant activity was assessed using in vitro DPPH and β-carotene assays, which showed a significant free radical scavenging capacity.

Subsequently, the antioxidant and anti-inflammatory effects were evaluated in LPS-stimulated RAW 264.7 macrophages. The extract was shown to reduce oxidative stress and protect DNA integrity, as demonstrated by DAPI staining, comet assay, and DNA fragmentation analyses. In addition, it decreased nitric oxide and malondialdehyde levels while increasing glutathione levels and regulating antioxidant enzyme activity. At the molecular level, inhibition of the expression of pro-inflammatory genes (iNOS, COX-2, TNF-α, IL-6, and IL-1β) was observed, together with a reduction in iNOS and COX-2 protein expression. Overall, the study suggested that the aqueous extract of *Glechoma hederacea* possesses antioxidant and anti-inflammatory potential owing to its phytochemical composition [12,118].

Taken together, the available experimental studies consistently demonstrate that *Glechoma hederacea* exhibits antioxidant, anti-inflammatory, antimicrobial, and cytoprotective properties. Nevertheless, the reported biological activities vary according to the extraction solvent, the plant material investigated, the phytochemical composition of the extracts, the analytical methods used for compound characterization, the experimental model employed, and the biological outcome measures, making direct comparisons among studies difficult. Future studies should also include standardized extracts characterized by defined concentrations of major bioactive constituents to improve the reproducibility and comparability of biological findings. Overall, the current evidence supports the pharmacological potential of the species, while emphasizing the need for standardized experimental protocols and further validation in clinically relevant models.

## 6. Applications of *Glechoma hederacea* in Oral Health

Despite the increasing interest in medicinal plants for oral healthcare, direct evidence regarding the application of *Glechoma hederacea* in dentistry remains limited. Consequently, the potential relevance of this species is currently supported by a combination of direct experimental observations, studies investigating isolated phytochemicals, and indirect evidence derived from non-oral disease models. This distinction should be considered when interpreting the available literature.

The adult oral cavity typically harbors more than 200 bacterial species, while over 600 species have been identified across the general population. Analyses based on 16S rRNA gene sequencing have demonstrated that complex microbial communities are already present by approximately four months of age, including genera commonly found in adults, such as *Streptococcus, Veillonella*, and *Neisseria* [119].

Additional studies investigating the oral microbiome in infants have revealed a lower microbial diversity compared with later stages of life and have highlighted the influence of factors such as maternal smoking, mode of delivery, and breastfeeding. Earlier culture-based studies examined the succession of colonization by key bacterial species, including *Streptococcus mitis* as an early colonizer and *Streptococcus sanguinis* as a species associated with later colonization. Oral diseases can impair mastication and reduce social interactions, and over time these consequences may contribute to nutritional deficiencies and psychosocial stress [120].

Research investigating colonization by the cariogenic pathogen *Streptococcus mutans* has yielded variable results. Some studies reported its presence only after tooth eruption, whereas others detected it even in predentate infants. Overall, culture-based studies have contributed substantially to the understanding of the stepwise development of early oral bacterial communities [100]. According to the 2022 report of the World Health Organization, approximately 3.5 billion people worldwide are affected by oral diseases. Among them, nearly 2 billion suffer from dental caries in the permanent dentition, while approximately 530 million children are affected by caries in the primary dentition [121]. Dental caries is a common infectious disease primarily associated with the activity of *Streptococcus mutans*, affecting a substantial proportion of the population. This bacterial species is considered the principal etiological agent of dental caries and is implicated in approximately 60–80% of cases in children and in the majority of cases in adults. *Streptococcus mutans* contributes to the development of oral infectious processes through the utilization of dietary sucrose for the synthesis of exopolysaccharides, which are essential components of dental plaque formation, adhesion to tooth surfaces, and organization of the extracellular matrix. These mechanisms promote the progression of carious lesions and may eventually lead to severe oral infections [122]. A key factor in the pathogenicity of this bacterium is its ability to form biofilms on host tissues, a critical step in the initiation and persistence of infection. Therefore, controlling *Streptococcus mutans* populations or inhibiting biofilm formation is considered an important strategy in the prevention of dental caries [123]. Natural products of plant origin, particularly those derived from medicinal plants, represent an important source of bioactive compounds with significant potential for the development of novel therapeutic and prophylactic agents in dentistry. In the context of increasing resistance of pathogenic microorganisms to conventional therapies, such as antibiotics and antivirals, interest in identifying natural antibacterial alternatives has grown considerably [124,125].

From an applied perspective, the exploitation of these plant-derived compounds is supported by the need to develop new natural dental products characterized by strong antimicrobial activity and the ability to inhibit bacterial biofilm formation, particularly that of *Streptococcus mutans* [126]. A crucial step in bacterial biofilm development is the adhesion of microorganisms to surfaces, whether dental or otherwise. Natural compounds derived from plant extracts may interfere with this process through several mechanisms. One mechanism involves anti-adhesive activity, whereby phenolic compounds, such as catechins from green tea, inhibit interactions between bacteria and receptors located on tissue surfaces. Consequently, the initial attachment capacity of *Streptococcus mutans* is reduced [127]. Another mechanism involves competition for adhesion sites. Certain plant extracts, particularly those derived from citrus fruits, may act as competitive agents by occupying available binding sites and thereby preventing bacterial adhesion to surfaces [128,129]. Numerous plant extracts also exhibit antioxidant activity, which may influence both bacterial resistance mechanisms and their ability to form biofilms [130].

At the mechanistic level, phenolic compounds, including polyphenols, exert antibacterial activity through multiple mechanisms, including disruption of bacterial cell membranes, alteration of membrane permeability, inhibition of essential enzymes, and interference with biofilm formation. These effects result in reduced bacterial viability and impaired biofilm development, thereby limiting bacterial survival and pathogenicity [131]. One of the principal groups of microorganisms present in the normal oral microbiota is the genus *Candida*, which consists of dimorphic commensal yeasts. Under physiological conditions, *Candida* species are generally non-pathogenic; however, when oral microbiome homeostasis is disrupted, they may become opportunistic pathogens and are frequently implicated in oral fungal infections [132]. Although specific experimental data regarding the antifungal activity of *Glechoma hederacea* against *Candida albicans* remain limited, the available literature suggests a potential inhibitory effect, primarily attributed to its high content of phenolic compounds, such as rosmarinic acid and various flavonoids. These bioactive constituents may interfere with processes essential for fungal pathogenicity, including adhesion, biofilm formation, and cellular viability. Studies conducted on other medicinal species belonging to the *Lamiaceae* family have reported significant antifungal and antibiofilm effects against *Candida albicans*, supporting the hypothesis that *Glechoma hederacea* may exhibit similar activities due to its comparable phytochemical profile. In this context, *Glechoma hederacea* may be considered a promising candidate for future investigations focused on the management of oral candidiasis and biofilm-associated fungal infections [133].

**Figure 3:**
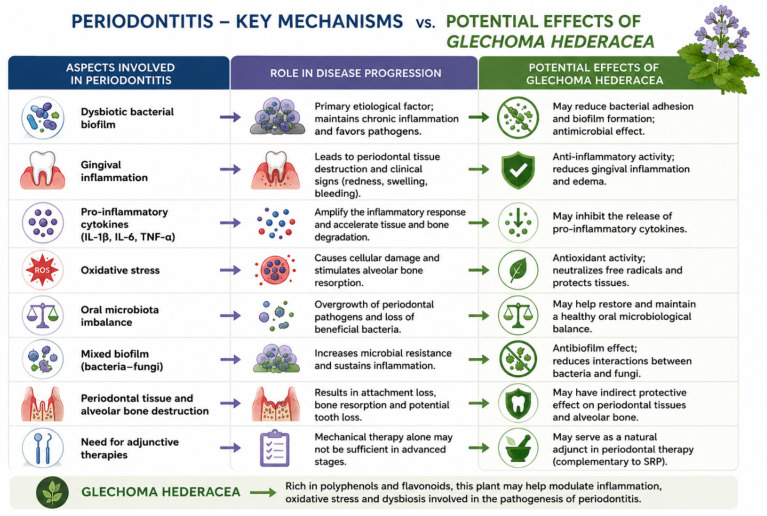
Schematic representation of the main mechanisms involved in the pathogenesis of periodontitis and the potential effects associated with the use of *Glechoma hederacea* [103,105,146,147].

Periodontal disease and cardiovascular diseases are highly prevalent conditions worldwide and share several interconnected pathophysiological mechanisms [134]. Periodontitis is a chronic inflammatory disease associated with the development of a dysbiotic microbial biofilm and persistent activation of the host immune response [135,136]. Disease progression is associated with the release of pro-inflammatory cytokines, including IL-1β, IL-6, and TNF-α, as well as increased oxidative stress, all of which contribute to periodontal tissue degradation and alveolar bone resorption [137]. Current evidence suggests that conventional mechanical therapy, consisting of scaling and root planing, may be enhanced by adjunctive therapies based on natural compounds with anti-inflammatory, antioxidant, and antimicrobial properties [138]. In this regard, medicinal plants belonging to the *Lamiaceae* family, recognized for their high content of phenolic compounds and flavonoids, have attracted growing interest in the management of periodontal diseases [139,140]. Due to its antioxidant, antimicrobial, and anti-inflammatory properties described in the literature [141], *Glechoma hederacea* may represent a promising source of bioactive compounds with the potential to modulate the periodontal inflammatory response and contribute to maintaining oral microbial homeostasis, as illustrated in Figure 3 [137,141], although these mechanisms remain hypothetical in the context of oral-specific applications and require experimental validation. The formation of dental biofilm is influenced from its earliest stages by several factors, including surface free energy, which determines wettability and surface tension properties, dental surface roughness, and the capacity for salivary protein adhesion. It is well established that oxidative stress plays an important role in cytotoxicity associated with chronic stress and contributes significantly to the progression of numerous diseases, including periodontitis [100,142].

Bioactive compounds have been reported to modulate apoptotic pathways in both host cells and pathogenic microorganisms, thereby playing an important role in maintaining cellular homeostasis and tissue integrity, particularly in chronic inflammatory conditions such as periodontal disease. Apoptosis, also known as programmed cell death, is a tightly regulated physiological process essential for the elimination of damaged or infected cells, thereby limiting tissue destruction and supporting healing and regenerative processes [143,144,145].

From an oral health perspective, the current literature emphasizes the essential role of microbial biofilms, oxidative stress, and persistent inflammatory responses in the development of dental caries, periodontal disease, and oral candidiasis. In this context, the use of bioactive compounds derived from *Glechoma hederacea* may represent a promising approach for the development of complementary phytotherapeutic strategies aimed at the prevention and management of oral diseases [6,9,147,148,149,150,151,152]. These proposed oral health applications are supported by the convergence of antioxidant, anti-inflammatory, and antimicrobial properties consistently reported in experimental studies, although direct oral evidence remains scarce.

Collectively, the currently available evidence supports the biological plausibility of *Glechoma hederacea* as a source of bioactive compounds for oral healthcare. However, this conclusion relies predominantly on indirect evidence derived from non-oral experimental models or studies investigating isolated phytochemicals. Consequently, the current evidence should be interpreted cautiously until validated by oral-specific experimental studies and well-designed clinical investigations. To the best of our knowledge, no clinical trials or human intervention studies evaluating the therapeutic efficacy of *Glechoma hederacea* have been published to date. Consequently, the current evidence is derived predominantly from in vitro studies, experimental animal models, and phytochemical investigations, highlighting the need for well-designed clinical studies before clinical recommendations can be established.

Regarding safety, although the available evidence suggests a favorable preliminary safety profile of *Glechoma hederacea* extracts, comprehensive toxicological evaluation remains limited. Available in vitro studies have reported no significant cytotoxic effects at the concentrations tested, supporting the biocompatibility of the investigated extracts under experimental conditions. Nevertheless, these findings are largely restricted to laboratory studies and cannot yet be extrapolated to clinical use. Furthermore, information regarding adverse effects, contraindications, and potential herb–drug interactions remains scarce in the current literature. As with other herbal medicinal products, the safety of *Glechoma hederacea* preparations may depend on factors such as the extraction method, phytochemical composition, dosage, duration of administration, and concomitant use with conventional medications. Therefore, further toxicological, pharmacokinetic, and well-designed clinical studies are required to establish the long-term safety of *Glechoma hederacea* before routine therapeutic application can be recommended.

As summarized in Table 4, direct evidence supporting the oral health applications of *Glechoma hederacea* remains limited, with most currently available data originating from non-oral experimental models. Therefore, although the reported antioxidant, anti-inflammatory, and antimicrobial properties provide a strong biological rationale for future oral applications, dedicated oral-specific studies and well-designed clinical trials are still required before definitive conclusions can be drawn.

## 7. Conclusions

The reviewed studies indicate that *Glechoma hederacea* extracts and several of their major phytochemical constituents exhibit significant antioxidant, anti-inflammatory, and antimicrobial activities. These biological effects have been associated primarily with phenolic compounds, including rosmarinic acid, caffeic acid, chlorogenic acid, and flavonoids identified in *Glechoma hederacea* extracts. Collectively, the available experimental evidence suggests that these properties may support the therapeutic potential of the species in conditions associated with oxidative stress, chronic inflammation, and microbiological imbalance, although the current evidence is derived predominantly from in vitro and experimental studies.

Despite the encouraging findings, interpretation of the available evidence should consider the considerable methodological heterogeneity among studies, including differences in extraction procedures, phytochemical characterization, experimental models, and biological outcome measures. These factors currently limit direct comparison between studies and reinforce the need for standardized research approaches.

The present review also has several limitations. The currently available evidence is based predominantly on in vitro studies, experimental animal models, and phytochemical investigations, while direct oral-specific studies and clinical trials remain scarce. In addition, differences in extraction procedures, phytochemical characterization, and experimental methodologies limit direct comparison among studies and preclude definitive conclusions regarding clinical efficacy.

Furthermore, the phytochemical profile of the species and the biological activities described in the reviewed studies support the possibility of developing pharmacological and phytotherapeutic products for oral health applications. *Glechoma hederacea* extracts could be incorporated into formulations such as mouthwashes, gingival gels, oral sprays, or other adjunctive products intended for the control of microbial biofilms and the reduction in oral inflammation. However, it should be noted that most of the available evidence originates primarily from in vitro and experimental studies, whereas clinical research investigating the use of this species in dentistry remains limited. Therefore, further studies are required to elucidate its mechanisms of action, standardize extract preparations, evaluate biocompatibility, and validate its clinical efficacy in oral therapeutic applications.

Although the available findings are encouraging, direct evidence supporting the application of *Glechoma hederacea* in oral healthcare remains limited, particularly with respect to oral-specific experimental models and clinical studies.

Future research should prioritize standardized oral in vitro models, animal studies, and clinical investigations to clarify the therapeutic potential of *Glechoma hederacea* in dentistry.

## Figures and Tables

**Table 3:** Biological activities of the principal phytochemical constituents identified in *Glechoma hederacea*.

Compound Class	Bioactive Compound	Reported Biological Activities	References
Phenolic acids	Rosmarinic acid	major compound; strong antioxidant and anti-inflammatory activity	[52,55,102]
	Caffeic acid	antioxidant activity; important metabolic precursor	[41,42]
	Chlorogenic acid	antioxidant effect and redox stabilization	[63,65]
	Ferulic acid	moderate antioxidant activity	[74,76]
	*p*-Coumaric acid	minor phenolic compound with antioxidant role	[82,84]
Flavonoids	Rutin	major flavonoid with antioxidant effect	[103]
	Quercetin derivatives	anti-inflammatory and antioxidant activity	[87]
	Apigenin derivatives	anti-inflammatory effect	[104,105]
	Luteolin derivatives	antioxidant and immunomodulatory activity	[106]
	Kaempferol derivatives	moderate antioxidant activity	[7]
Triterpenoids	Ursolic acid	anti-inflammatory and antimicrobial activity	[107,108]
	Oleanolic acid	anti-inflammatory and cytoprotective effect	[107,109]

**Table 4:** Summary of the available direct evidence regarding the oral health-related effects of *Glechoma hederacea*.

Oral Health Aspect	Direct Evidence for *Glechoma hederacea*	Main Source of Current Evidence
Antibacterial activity against oral pathogens	Very limited	Non-oral in vitro studies
Antibiofilm activity	Very limited	Studies on plant phenolics and related species
Antifungal activity (*Candida albicans*)	No direct oral studies	Related *Lamiaceae* species and non-oral models
Anti-inflammatory effects in periodontal disease	No oral-specific studies	General pharmacological studies
Clinical application	No clinical studies identified	Future research needed

## Data Availability

The original contributions presented in the study are included in the article. Further inquiries can be directed to the corresponding author.

## Abbreviations

The following abbreviations are used in this manuscript:

CO_2_	Carbon dioxide
COX-2	Cyclooxygenase-2
DAPI	4′,6-Diamidino-2-phenylindole
DNA	Deoxyribonucleic acid
DPPH	2,2-Diphenyl-1-picrylhydrazyl
HWG	Hot-water extract of *Glechoma hederacea*
IL-1β	Interleukin-1 beta
IL-6	Interleukin-6
iNOS	Inducible nitric oxide synthase
LPS	Lipopolysaccharide
NFATc1	Nuclear factor of activated T cells 1
PI3K/AKT	Phosphoinositide 3-kinase/Protein kinase B signaling pathway
PRISMA	Preferred Reporting Items for Systematic Reviews and Meta-Analyses
TNF-α	Tumor necrosis factor alpha
